# Regulation of mRNA Trafficking by Nuclear Pore Complexes

**DOI:** 10.3390/genes5030767

**Published:** 2014-09-02

**Authors:** Amandine Bonnet, Benoit Palancade

**Affiliations:** Institut Jacques Monod, CNRS, UMR 7592, University Paris Diderot, Sorbonne Paris Cité, Paris F-75205, France; E-Mail: bonnet.amandine@ijm.univ-paris-diderot.fr

**Keywords:** mRNA export, mRNA quality control, nuclear pore complexes (NPCs), nucleoporin, post-translational modifications

## Abstract

Over the last two decades, multiple studies have explored the mechanisms governing mRNA export out of the nucleus, a crucial step in eukaryotic gene expression. During transcription and processing, mRNAs are assembled into messenger ribonucleoparticles (mRNPs). mRNPs are then exported through nuclear pore complexes (NPCs), which are large multiprotein assemblies made of several copies of a limited number of nucleoporins. A considerable effort has been put into the dissection of mRNA export through NPCs at both cellular and molecular levels, revealing the conserved contributions of a subset of nucleoporins in this process, from yeast to vertebrates. Several reports have also demonstrated the ability of NPCs to sort out properly-processed mRNPs for entry into the nuclear export pathway. Importantly, changes in mRNA export have been associated with post-translational modifications of nucleoporins or changes in NPC composition, depending on cell cycle progression, development or exposure to stress. How NPC modifications also impact on cellular mRNA export in disease situations, notably upon viral infection, is discussed.

## 1. Introduction

**Figure 1:**
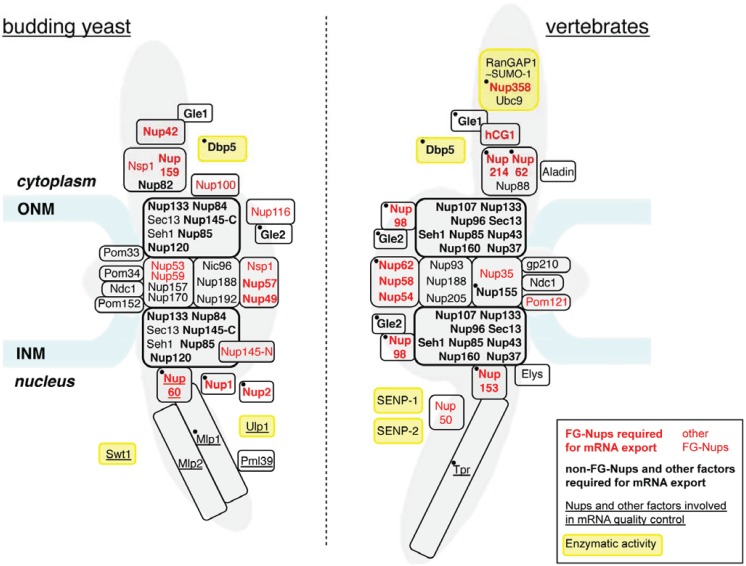
Nucleoporins and nuclear pore complex (NPC)-associated proteins involved in mRNA export. The approximate relative positioning of nucleoporins or NPC subcomplexes within the NPC framework is represented in budding yeast (left) and vertebrates (right) (according to [10]). Phenylalanine-glycine (FG)-nucleoporins appear in red. Nucleoporins (Nups) or NPC-associated proteins with a reported contribution to mRNA export are indicated in bold (see Table 1). The names of proteins involved in mRNA quality control are underlined (see Table 2). Factors targeted by regulatory events occurring in normal or pathological situations and mentioned in the text (Section 4 and Section 5) are indicated by a black dot. Proteins carrying an enzymatic activity are boxed in yellow. Alternative names for vertebrate nucleoporins are the following: Nup358 = RanBP2; Gle2 = Rae1; Nup35 = Nup53; Nup58 = Nup45; Elys = MEL28; hCG1 = NPL1. The Y-complex is boxed by a thick black line. Note that the inactivation of each Y-complex subunit has not systematically been proven to trigger mRNA export defects: in yeast, *seh1* and *sec13* mutants do not affect mRNA export [11]; in mammals, mRNA export inhibition has solely been reported upon Nup133 or Nup107 siRNA-mediated depletion [12,13] or upon expression of dominant negative fragments of Nup133 or Nup160 [14]. ONM, outer nuclear membrane; INM, inner nuclear membrane.

One of the defining hallmarks of eukaryotic cells is the compartmentalization of their genome, which enables the fine-tuning of gene expression processes, from mRNA synthesis and processing in the nucleus to translation in the cytoplasm. Exchanges of molecules between the two compartments exclusively rely on nuclear pore complexes (NPCs), which are multiprotein assemblies composed of multiple copies of ~30 different nucleoporins (Nups; Figure 1) and whose estimated mass is 60 MDa in yeast and 125 MDa in vertebrates. The overall NPC structure is conserved from yeast to human and is organized according to an eight-fold symmetry, encompassing a stable membrane-embedded scaffold, which delineates a ~40-nm central channel and anchors peripheral structures, namely the nuclear basket and the cytoplasmic filaments [1,2]. A selective barrier to the diffusion through the NPC channel is established by the interaction of several unstructured and hydrophobic FG-domains, composed of multiple clusters of the phenylalanine-glycine (FG) dipeptide separated by hydrophilic linkers [3]. The meshwork thus formed enables either the passive diffusion of small molecules or the selective transport of larger molecules (proteins and ribonucleoparticles) harboring nuclear localization or export signals (NLS/NES). These sequences are recognized by transport receptors (importins/exportins), which can dynamically interact with FG repeats [3]. The directionality and the irreversibility of the diverse protein and RNA transport pathways are provided by the regulated formation and dissociation of transport complexes on both sides of the NPCs [1,2].

In the case of mRNAs, a pre-requisite for nuclear export is the formation of messenger ribonucleoparticles (mRNPs), which encompass multiple proteins transferred to the transcripts during the course of transcription, splicing and 3'-end processing. Acquisition of export competence is associated with the completion of pre-mRNA processing reactions, the release of packaged mRNPs from the transcription site and the recruitment of mRNA export receptors that will ultimately interact with FG-nucleoporins at NPCs. In yeast, the unique mRNA export receptor is the Mex67-Mtr2 heterodimer, whose association with mRNAs requires dedicated RNA-binding adaptors, such as the Yra1 subunit of the TREX (transcription and export) complex, Nab2 or Npl3 [4,5,6]. The situation appears somehow more complex in metazoans, with the coexistence of two mRNA export pathways. The majority of mRNAs are exported by virtue of their interaction with the orthologue of the Mex67-Mtr2 heterodimer (called Tap-p15 or NXF1-NXT1 in vertebrates), whose association to mRNAs is facilitated by the conserved TREX complex. In contrast, the export of a subset of mRNPs depends on the exportin Crm1, which recognizes distinct NES-containing adaptors, such as NXF3 or LRPPRC, bound to specific cis-acting mRNA elements [7,8].

Recent reviews have paid attention to the molecular mechanisms underlying mRNP assembly and export and to their tight connections with mRNA transcription, processing and nuclear organization, in yeast and/or in metazoans [4,5,6,7,8,9]. The present review will be focused on the role of NPCs in mRNA export from yeast to human. We will first briefly review the known function of FG-domains, scaffold nucleoporins and NPC-associated proteins in constitutive mRNA export. We will then summarize our current knowledge on the role of NPC-associated proteins in mRNA quality control prior to export, an expanding field of research. Finally, we will show how regulatory mechanisms targeting NPCs have been proposed to interfere with mRNA export during cell cycle and development or in stress and disease situations.

## 2. Role of NPC Components in Constitutive mRNA Export

Multiple approaches have been used to characterize the main steps of mRNA export through NPCs and the nucleoporins involved in this process. Genetic screens performed in yeast, followed by microscopy detection of poly(A) RNAs by fluorescence *in situ* hybridization (FISH) in mutants, have been instrumental in identifying most of the nucleoporins contributing to mRNA export [15,16] (Table 1). Imaging in large metazoan cells has further made it possible to gain access to the choreography of mRNP transport through NPCs [5,17]. Early electron microscopy studies had visualized the interactions between the NPC and a large mRNP in the dipteran *Chironomus* [18,19]. More recently, live tracking of single mRNPs, individually labeled with fluorophores, revealed that nuclear export of mRNAs through NPCs can be divided into three sequential steps: docking of the mRNP at the nucleoplasmic face, transport through the pore channel and release from the cytoplasmic face [20,21,22].

### 2.1. FG-Nucleoporins Are Critical for mRNP Transport

The low-affinity interactions between transport receptors and distinct classes of FG-domains found in central and peripheral nucleoporins are the basis for all of the proposed models of translocation [2,3]. Consistently, FG-nucleoporins play a pivotal role in mRNA export through their conserved interaction with mRNA export receptors (Figure 1). The export receptors, Mex67-Mtr2 and Tap-p15, respectively interact with most yeast and vertebrate FG-nucleoporins *in vitro*, and this interaction is critical for their recruitment at NPCs and, subsequently, for mRNA export *in vivo* [23,24,25,26,27,28].

**Table 1:** Nucleoporins involved in mRNA export in yeast.

		Phenotypes Observed upon Inactivation		
	FG Repeats	Viability	mRNA Export	References
**Symmetric Nups**				
Nup53	FG	V	*n.d.*	
Nup59	FG	V	*n.d.*	
Nup157		V	+	
Nup170		V	+	
Nup133		ts	−	[32,33]
Nup84		ts	−	[11]
Nup145-C		ts	−	[34,35,36]
Nup85		ts	−	[37]
Nup120		ts	−	[38,39]
Sec13		L ^a^	+	
Seh1		cs	+	
Nic96		L	+	
Nup188		V	+	
Nup192		L	+	
Nsp1	FG, FxFG	L	+	
Nup57	GLFG	L	− ^b^	[28]
Nup49	GLFG	L	−	[28,32]
Nup145-N	GLFG	V	+	
Nup116	FG, GLFG	L/ts ^e^	− ^c^	[31]
Gle2		ts	−	[40]
Nup100	GLFG	V	+	
Ndc1		L	*n.d.*	
Pom34		V	*n.d.*	
Pom152		V	+	
Pom33		V	+	
**Asymmetric Nups**				
Nup82		L	−	[41,42]
Nup159	FG	L	−	[43]
Nup42	FG	V	− ^d^	[44,45,46]
Gle1		L	−	[47]
Dbp5		L	−	[48,49]
Nup60	FxF	V	−	[50]
Nup1	FxFG	L/ts ^e^	−	[28,51,52]
Nup2	FxFG	V	− ^b^	[28]
Mlp1		V	+	
Mlp2		V	+	

The types of FG-repeats [29], as well as the growth phenotypes observed upon inactivation of each yeast nucleoporin [16,30] are indicated: V, viable; L, lethal; ts, thermosensitive; cs, cold-sensitive. mRNA export phenotypes have been assayed using deletion mutants (for non-essential nucleoporins) or truncation or conditional alleles (for essential nucleoporins): (+), normal mRNA export in the corresponding mutant; (−), mRNA export defects reported in the mutant; *n.d.*, not determined. ^a^ Sec13 essentiality may be related to its function in the essential process of secretion. ^b^ In this case, the effect on mRNA export has only been reported in the context of combined FG deletions [28]. ^c^ While the *nup116* deletion mutant exhibits mRNA export defects, most likely caused by nuclear envelope abnormalities, deletion of Nup116 glycine-leucine-phenylalanine-glycine (GLFG) repeats does not trigger mRNA retention. ^d^ Nup42 is required for mRNA export under heat shock conditions, but is dispensable for mRNA export under normal growth conditions. ^e^ Nup116 and Nup1 essentiality depends on the genetic background [16,30,31].

The detailed analysis of the contribution of each FG-nucleoporin to mRNA export was further genetically dissected through combined deletion of FG-domains in yeast [28]. Central FG-repeats, which typically encompass GLFG motifs, were found to be essential for viability, while peripheral FG-repeats are dispensable for growth [28,29]. From these studies, four FG-nucleoporins have emerged as key players for efficient Mex67 recruitment at NPCs and mRNA export: Nup49, Nup57, Nup1 and Nup2 [28]. The corresponding FG-domains appear to be redundant, since none of them is individually required for mRNA export, and are somehow pathway-specific, since distinct FG-domains are required for protein import [28,29]. In view of their localization at the nuclear basket (Nup1, Nup2) and within the central channel (Nup49, Nup57), these FG-nucleoporins are likely to be the main players in mRNP docking and translocation, respectively. Orthologue components within the vertebrate NPC were also shown to contribute to mRNA export: Nup153, an FG-nucleoporin of the nuclear basket that shares certain features with yeast Nup1 [53,54], and the Nup62 complex [27,55], which is the orthologue of the yeast Nup57-Nup49-Nsp1 complex (Figure 1), participate in mRNP docking and translocation, respectively. In addition, other vertebrate FG-nucleoporins, such as Nup98 (complexed with Gle2/Rae1), also contribute to mRNA export [56,57].

At the cytoplasmic face of the NPC, the FG-domains of Nup159 and Nup42, albeit not essential for mRNA export, contribute to this process by positioning mRNPs for remodeling steps in yeast (see below, Section 2.3) [58]. Strikingly, FG “swap” experiments, replacing these cytoplasmic FG repeats with repeats of other FG-nucleoporins, demonstrated that all FG motifs are not functionally equivalent [58]. In metazoans, mRNA export defects have also been observed in the absence of two FG-nucleoporins of the cytoplasmic face of NPCs, namely Nup214 [59] and Nup358/RanBP2 [60].

### 2.2. Non-FG-Nucleoporins May Provide Additional mRNP Binding Sites

In addition to FG-nucleoporins, genetic approaches also identified other nucleoporins as required for mRNA export in yeast, most of them being components of the Nup84 complex (also referred to as Y-complex; Figure 1) [16,61]. Indeed, inactivation of Nup84, Nup85, Nup120, Nup133 or Nup145-C similarly leads to mRNA export defects, without any detectable consequences on protein transport [11,32,33,34,35,37,38,39]. These mutations were also found to trigger NPC clustering, a phenotype that can however be uncoupled from mRNA export defects in some specific *nup133* and *nup85* mutant alleles [32,62]. Importantly, the function of the Y-complex in mRNA export is conserved in vertebrates and plants [61]. In particular, expression of dominant negative fragments of Nup133 or Nup160 [14], or knockdown of Nup107 or Nup133, which impairs NPC assembly [12,13], inhibits mRNA export in HeLa cells. While the Y-complex could contribute to mRNA export through its function as an essential building block of the NPC scaffold, it could also provide additional binding sites for mRNPs at NPCs. Indeed, the Y-complex was shown to directly interact with Mex67-Mtr2 through its Nup85 subunit in yeast [62,63].

Interestingly, structural studies or fold assignment have identified RNA binding motifs within non-FG domains in mammalian Nup153 [64], Nup35 [65] and RanBP2 [66] and in yeast Nup53 and Nup59 [67]. Since these motifs could also serve protein-protein interaction purposes, their involvement in mRNA export *in vivo* awaits further validation. An RNA binding surface has also been characterized in the human Nup98-Gle2 complex [68]. The function of this region in mRNA export is further supported by the reported interaction between Gle2 and poly(A) RNAs in cross-linking experiments in HeLa cells [69] and by the mRNA retention phenotypes caused by Gle2 inactivation, both in yeast and vertebrate cells [40,70,71].

### 2.3. NPC-Associated Factors Control mRNP Docking and Release

At the nucleoplasmic face of NPCs, multiple non-FG factors contribute to mRNP docking prior to export, including the nuclear basket proteins, Mlp1 and Mlp2 (myosin-like proteins), in yeast (see Section 3 and Figure 1). In addition, the conserved TREX2 (transcription and export) complex couples transcription with mRNA export at NPCs [50,72,73,74]. In yeast, the Sac3 subunit of TREX2 interacts with Nup1 at NPCs via its C-terminal domain and with Mex67-Mtr2 via its N-terminal domain, which contains degenerate FG repeats [50], therefore providing an additional mRNP binding site at the nuclear basket.

mRNP release, a critical step in mRNA export, occurs at the cytoplasmic side of NPCs. Studies from several groups have highlighted an essential function in this process performed by Dbp5 (also called DDX19 in mammals), a conserved DEAD-box ATP-dependent RNA helicase associated with NPC cytoplasmic filaments and required for mRNA export [48,49,75]. Nucleotide-dependent conformational changes of Dbp5 were shown to promote mRNP remodeling events, leading to the displacement of Mex67 and Nab2 from mRNPs in yeast [76,77], thus ensuring the irreversibility of the mRNA transport process. Directionality is further provided by the local activation of Dbp5 at the cytoplasmic side of NPCs. Indeed, the ATP-ADP nucleotide cycle and the remodeling activity of Dbp5 are controlled by two components of the cytoplasmic filaments, Gle1, bound to its cofactor, inositol hexakisphosphate (IP6) [78,79], and the N-terminal non-FG domain of Nup159 (Nup214 in mammals) [80,81,82]. Besides these two factors, this process requires in yeast: (i) Nup82, a non-FG-Nup anchoring Nup159 to the NPC scaffold; (ii) Nup42, which tethers Gle1 to the cytoplasmic filaments; and (iii) the Nup159 and Nup42 FG-domains that position Mex67-bound mRNPs in close vicinity of Dbp5 [58]. Multiple factors therefore contribute to the termination of mRNP export at the cytoplasmic face of NPCs (Figure 1), and this system is remarkably conserved from yeast to metazoans [75,80,83].

## 3. mRNA Quality Control at the Nuclear Basket of NPCs

mRNA quality control (QC) designates the mechanisms by which cells discard incompletely processed or improperly assembled mRNPs, avoiding their accumulation, which would be detrimental for protein homeostasis [84]. Although QC is mainly ensured by nuclear and cytoplasmic RNA degradation activities, several reports have indicated that proteins anchored at the nucleoplasmic side of NPCs contribute to a QC step prior to mRNA export. This function of the nuclear basket was initially inferred from studies of its constituents, Mlp1-2, in yeast or its orthologue, Tpr (translocated promoter region), in mammals. While inactivation of these proteins does not affect bulk mRNA export ([85,86] and the references therein), their overexpression results in nuclear accumulation of poly(A) RNAs, both in yeast and mammalian cells [85,87]. At that time, this observation was interpreted as a possible consequence of the titration of factors necessary for efficient mRNA export. This relationship with mRNA export was further confirmed, initially in yeast and, more recently, in mammals.

### 3.1. Mlp1-2, Central Players in mRNA QC in Yeast

Multiple physical interactions have been identified between Mlp1-2 and mRNPs: beside a direct, well-characterized interaction between Mlp1 and the RNA-binding adaptor, Nab2 [88], recent proteomic analyses have revealed that Mlp1 and Mlp2 interactomes encompass multiple mRNP components [89,90]. These data imply that nuclear basket proteins could be primarily involved in the docking of mRNPs at the nuclear side of NPCs prior to their translocation. Consistent with these findings, specific abolition of the Mlp1-Nab2 interaction diminishes mRNA export efficiency [91]. While this last result suggests that Mlp1 would select Nab2-bound mRNPs and commit them for translocation, there is also evidence that Mlps can specifically anchor and retain improperly assembled mRNPs. Indeed, a persistent association was observed between Mlp2 and a mutant version of the Yra1 adaptor that appears unable to load the Mex67 export receptor onto mRNPs (*GFP-yra1-8*) [92]. The rescue of the growth defects of yeast *yra1* or *nab2* mRNP assembly mutants upon loss of Mlp1/2 [92] further supports the idea that misassembled mRNPs would detrimentally get trapped onto the Mlp1/2 platform and released for export in their absence.

Docking mRNPs to the nuclear basket would not only monitor mRNP composition, but could also check the full completion of mRNA processing reactions. Since Nab2 is a poly(A)-binding protein required for proper poly(A) tail length [93], Mlp1-Nab2 interaction could specifically select the mRNA molecules that have undergone 3' processing. Mlp1 was also proposed to play a role in mRNA QC following splicing. A synthetic lethal screen using *MLP1* inactivation as a bait identified a mutant of the splicing factor Prp18, suggesting that Mlp1 function is essential when splicing is sub-optimal [94]. Using a dedicated LacZ reporter gene, it was further shown that *MLP1* deletion triggers the cytoplasmic leakage of intron-containing pre-mRNAs with no detectable effect on their splicing efficiency. Conversely, *MLP1* overexpression specifically traps mRNPs issued from an intron-containing reporter in the nucleus [94]. Similarly, overexpression of Nup211, the *S. pombe* homolog of Mlp1, decreases cytoplasmic leakage of pre-mRNAs accumulating upon pharmacological inhibition of splicing [95]. The QC function of Mlps, therefore, extends to the detection of splicing completion, although the mechanism of recognition of spliced mRNAs remains to be deciphered.

### 3.2. Multiple NPC-Associated Factors Contribute to mRNA QC in Yeast

Following these first reports, a number of nuclear pore-associated proteins were shown to contribute to NPC-associated QC in yeast (Table 2). Their contribution to QC was mainly scored using the aforementioned leakage reporter system; however, nuclear mRNP retention upon overexpression and genetic interactions with mutants of mRNP components have also been reported in some cases (Table 2). Remarkably, some of these factors (namely Mlp1-2, Pml39, Ulp1) are excluded from the NPCs present in the nucleolus-proximal region of the nuclear envelope, possibly reflecting their intimate connection with mRNP export [94,96,97].

mRNAs targeted by QC mechanisms are ultimately discarded by degradation activities. In this respect, NPC-associated QC has been reported to involve the Swt1 endonuclease, a fraction of which could transiently associate with NPCs, where it has been detected in specific mutant contexts [98]. Other degradation activities, such as the nuclear exosome, might also participate in this process.

NPC-associated QC has also been proposed to require specific post-translational modification events. Remodeling of mRNPs prior to export has been shown to involve ubiquitination-induced dissociation of Yra1 [99]. Since the growth defects of non-ubiquitinable mutants of Yra1 are suppressed upon *MLP1-2* deletion, this post-translational modification could contribute to the release of mRNPs from their docking sites at the nuclear basket. The SUMO-protease Ulp1 was also reported to prevent apparent pre-mRNA leakage, although QC-specific SUMOylated targets await identification [100].

Finally, it is important to point out that a number of non-NPC proteins were proposed to contribute to NPC-associated QC [101,102]. In particular, the SR-like proteins Hrb1 and Gbp2 have been proposed to mark properly spliced mRNPs and to commit them for export, possibly in relation to Mlp1 [102].

**Table 2:** NPC-associated proteins involved in mRNA QC prior to export in yeast. The proteins identified as contributing to mRNA QC in yeast are presented. QC phenotypes arising upon inactivation or overexpression of the protein are listed. The detection of leakage phenotypes with the LacZ-based reporter system (“pre-mRNA leakage”, (+)), as well as the names of the mRNP mutants that are rescued upon inactivation of the protein, are shown.

	Localization	Molecular function	Inactivation		Overexpression	
			Pre-mRNA Leakage	mRNP Mutants Rescued	Nuclear mRNP Accumulation	References
Mlp1	Nuclear basket ^a^	mRNP docking	+	*∆N-nab2*	+ ^d,e^	[85,92,94,99]
				*yra1-KR*		
Mlp2	Nuclear basket ^a^	mRNP docking	−	*∆N-nab2*	− ^d^	[85,92,94,99]
				*yra1-8*		
				*yra1-KR*		
				*tom1∆*		
Pml39	Nuclear basket ^a^	mRNP docking ?	+	*yra1-8*	+ ^e,f^	[96]
				*∆N-nab2*		
Nup60	NPC	Nuclear basket assembly ?	+	*n.d.*	*n.d.*	[94]
Esc1	Inner nuclear membrane- associated	Nuclear basket assembly ?	+	*n.d.*	*n.d.*	[100]
Ulp1	NPC ^a,b^	SUMO deconjugation	+	*n.d.*	*n.d.*	[100]
Swt1	NPC ^c^	RNA degradation	+	*n.d.*	+ ^d^	[98]

Accumulation of mRNPs in the nucleus upon overexpression of the protein is indicated by a (+). *n.d.*, not determined. ^a^ For these proteins, an asymmetrical localization at the nuclear periphery, excluded from the NPCs adjacent to the nucleolus, has been reported. ^b^ NPC localization of Ulp1 depends on several molecular determinants, including Nup60/Mlp1-2, the Y-complex and karyopherins [80]. ^c^ NPC localization of Swt1 has only been observed in specific mutant contexts [98]. ^d^ In this case, nuclear mRNA accumulation has been scored by FISH-based detection of poly(A) RNAs. ^e^ In this case, nuclear mRNA accumulation has been scored by FISH-based detection of LacZ mRNAs. ^f^ In this case, nuclear mRNP accumulation has been scored by Nab2 localization. Note that Tpr, the Mlp1-2 mammalian orthologue, is the only protein reported to function in a similar pathway in mammals. “?”, putative molecular function.

### 3.3. mRNA QC at Nuclear Pores: A Conserved Pathway in Mammalian Cells?

While NPC-associated QC has been essentially characterized using the budding yeast model, its conservation has been recently highlighted in mammalian cells, in particular through the study of viral mRNA export. Some retroviruses, for instance the MPMV (Mason-Pfizer monkey virus), are known to export RNAs with retained introns through a dedicated cis-acting RNA element referred to as CTE (constitutive transport element), which has the ability to directly recruit the Tap mRNA export receptor (the human orthologue of yeast Mex67). Other retroviruses, such as HIV (human immunodeficiency virus), export their unspliced RNAs using the RRE (Rev-responsive element), a RNA structure that recruits multiple copies of the NES-containing viral protein Rev, allowing mRNA export through the Crm1 pathway (reviewed in [103]). In recent studies, inactivation of Tpr, the Mlp1-2 human orthologue, was shown to increase the cytoplasmic localization and the translation of the intron-retaining form of a retrovirus-derived reporter RNA in a CTE-dependent manner [86,104]. Similar results were obtained with a non-viral CTE-containing reporter, but not with an RRE-containing construct, highlighting the specificity of this Tpr-dependent pathway for mRNA trafficking through Tap. Importantly, inactivation of Nup153, a nucleoporin previously shown to tether Tpr at the NPC [105], or expression of a Tpr mutant unable to interact with Nup153, similarly triggered the leakage of intron-retaining RNAs into the cytoplasm. Localization of Tpr at NPCs is therefore critical for its function in intron-containing mRNA retention [86].

It is noteworthy that the detection of mRNA QC phenotypes upon inactivation of nuclear pore components has mainly relied on the utilization of dedicated reporter systems, both in yeast and mammalian models. This raises the question of whether NPC-associated QC is biologically significant, eventually under specific physiological situations, and targets endogenous mRNAs. A recent report elegantly addressed this question, pointing to the potential importance of Tpr for the developmentally-programmed nuclear retention of a subset of mRNAs [106]. During neuron formation, a defined set of neuron-specific mRNAs, such as *STX1b*, undergoes regulated splicing, export and translation, while in non-neuronal cells expressing the splicing regulator, Ptbp1, these same mRNAs are improperly spliced, retained in the nucleus and degraded in a Tpr-dependent manner [106]. This example shows how NPC-associated QC could contribute to the control of gene expression programs in specific situations; whether additional examples of regulation via similar mechanisms exist deserves future investigations.

## 4. Role of NPCs in the Control of mRNA Export during Cell Cycle, Development and Stress

Transcriptional and post-transcriptional mechanisms are known to modulate the expression of cell cycle- and lineage-specific genes, thereby playing a key role in cell cycle progression, development and differentiation. During response to stress, the activation of specific signaling pathways also leads to changes in protein production, including the induction of molecular chaperones, which are essential for adaptation. Increasing evidence suggests that nucleoporins could contribute to these diverse regulatory events by controlling mRNA trafficking.

### 4.1. Modifications of NPCs and Cell Cycle-Dependent Changes in mRNA Export

In mammals, cell cycle-driven changes in mRNA export have been associated with oscillations of the levels of the nucleoporin Nup96, a subunit of the Y-complex. During mitosis (M), Nup96 is modified by ubiquitination and subsequently degraded by the proteasome. Interestingly, decreased levels of Nup96, as observed in M and G1, are associated with specific changes in the nuclear export of a subset of mRNAs, some of them encoding cell cycle regulators involved in the G1/S transition [107]. A post-translational modification targeting a unique nucleoporin can therefore regulate the nuclear export of a limited number of target mRNAs, thereby coordinating cell cycle progression. This generic NPC subunit could control mRNA export through direct interactions with specific mRNA-associated factors, either at the NPC or in the nucleoplasm, as proposed in [108]. Furthermore, this function of Nup96, and possibly additional regulatory mechanisms, could account for the global changes in bulk mRNA export observed during the cell cycle, in view of the decreased mRNA export detected in G1 as compared to G2 human cultured cells [107].

Nucleoporins, including Y-complex subunits, are modified by other post-translational modifications, in particular cell cycle-dependent phosphorylation [109]. While nucleoporin phosphorylation has been demonstrated to contribute to NPC disassembly or to modulate protein transport (see, for instance, [110,111]), it has not been shown to influence mRNA trafficking. However, Cdk1-dependent phosphorylation of Nup1 is required in budding yeast for the localization of some active genes at the nuclear periphery, a process known as gene gating [112]. Dephosphorylation of Nup1 has been proposed to account for the loss of peripheral localization observed for these genes during the S-phase. This regulatory mechanism could drive cell cycle-dependent changes in mRNA trafficking, since gene gating has been proposed to couple transcription with mRNA export at NPCs [112]. The multiple connections previously identified between NPCs and gene expression [2,113] could represent a target for similar regulations.

### 4.2. NPCs and Cell Cycle-Dependent Cytoplasmic Targeting of mRNAs

NPCs are not only important for the nuclear export of mRNAs, but have also important roles in their cytoplasmic localization following export. Such connections were observed early during flagellation in the green algae, *Chlamydomonas reinhardtii*, when NPC polarization to the posterior side of the nucleus is associated with the asymmetrical localization of the β2-tubulin transcript in the cytoplasm [114]. Asymmetrical mRNA localization has also been studied in budding yeast. The best characterized example is the mating-type switching regulator Ash1, whose mRNA is specifically localized to the daughter cell prior to cytokinesis. Strikingly, a subset of mRNAs that, similar to *ASH1*, are regulated during the cell cycle and exhibit asymmetrical localization in the cytoplasm, specifically require the nucleoporin Nup60 for their export out of the nucleus and their cytoplasmic targeting [115]. While this function of Nup60 in the fate of the mRNA remains to be understood, it provides an additional example of a crosstalk between nuclear basket proteins and mRNA metabolism.

### 4.3. NPC-Associated Proteins and mRNA Export during Development

Several analyses have revealed that NPC-associated proteins contributing to mRNA export are important for developmental processes. In plants, a number of mutants affecting nucleoporins or NPC-associated factors (including Y-complex, Mlps/Tpr and Ulp1 orthologues) exhibit poly(A) RNA export defects in association with multiple physiological or developmental abnormalities (reviewed in [116]). Plant mutants of the Y-complex also display altered levels for mRNAs encoding core circadian clock components, further supporting the notion that NPCs could contribute to the regulation of mRNA export and stability for a defined subset of mRNAs [117]. Of note, Y-complex mutations also lead to mRNA export defects in association with developmental deficiencies in metazoans. Nup96+/− heterozygous mice display alterations of the immune system and increased susceptibility to viral infections in association with defined mRNA export defects in immune cells [118]. In these different situations, it remains to be demonstrated whether abnormal export of specific mRNAs would be the unique cause of the developmental defects.

### 4.4. NPCs and Regulation of mRNA Export upon Stress

It is known that upon heat shock (HS), the export of heat shock protein (HSP)-encoding mRNAs occurs efficiently, favoring chaperone production, while poly(A) non-HSP mRNAs are specifically retained within the nucleus, both in yeast and mammalian cells [119,120]. Although in yeast, the HSP mRNA export process involves the core mRNA export machinery, including Mex67 and NPC components [46,119], it depends on fewer factors than bulk mRNA export [121], suggesting that it could be more resistant to stress-induced alterations of export activities.

Recently, the MAP kinase Slt2 was found to be critical for HS-induced retention of non-HSP mRNAs in yeast [122], suggesting the existence of phosphorylation events, either within mRNPs or at NPCs, that could trigger export inhibition. In addition, during HS, Mlp1 leaves the NPCs and forms intranuclear foci that sequester some mRNA-binding proteins, possibly affecting their recruitment onto mRNAs [122]. Identifying which signaling events ultimately lead to Mlp1 dissociation from NPCs will be required to refine our understanding of these processes. Notably, its mammalian orthologue, Tpr, is phosphorylated by several kinases, including MAP kinases [123], although the functional impact of these modifications on mRNA export remains to be determined. Tpr is also functionally important for HSP mRNA export through its interaction with the HS-induced transcriptional activator HSF1 [124]. The mechanisms used to favor HSP mRNA export at NPCs, hence, differ between species: in yeast, dissociation of the nuclear basket component Mlp1 may possibly inhibit non-HSP mRNA export, while docking of HSF1-bound HSP genes at the Tpr/nuclear basket would favor HSP mRNA export in mammals.

Other stress situations have been shown to target mRNA export at NPCs in yeast, either by triggering nucleoporin phosphorylation or by altering NPC composition. Phosphorylation of Nup1, Nup2 and Nup60 by the stress-activated kinase, Hog1, occurs upon osmotic stress, and these modifications have been proposed to contribute to optimal expression and export of stress-inducible mRNAs [125]. Other studies report that Gle2 and Dbp5 dissociate from NPCs in different stress conditions leading to inhibition of mRNA export [126,127]. In this last case, however, it remains to be determined how exposure to stress ultimately influences NPC composition.

## 5. NPCs and Dysregulation of mRNA Export in Disease Situations

In agreement with their key role at the interface between the nucleus and the cytoplasm, several NPC components have been identified as modified in different pathological situations, including genetic diseases, cancers and viral infections [103,128,129,130]. Since the integrity of the mRNA export process has not been investigated systematically in all of these situations, we will focus here on the cases for which mRNA export defects have been reported.

### 5.1. Alteration of the NPC Scaffold and Dysregulation of mRNA Export

Only a few non-FG-nucleoporins have been associated with diseases. Mutation of a central scaffold nucleoporin, Nup155, is responsible for a cardiac disorder referred to as atrial fibrillation (AF) [131]. Strikingly, both the missense Nup155 mutation found in AF, that affects Nup155 targeting to NPCs, and a reduction of Nup155 levels trigger a specific inhibition of *HSP70* mRNA export [131]. AF-associated mRNA export defects might be actually caused by improper localization of Gle1. Indeed, nuclear retention of *HSP70* mRNAs occurs in association with Gle1 delocalization upon knockdown of hCG1, which interacts with Nup155 and Gle1 [132]. However, to what extent these molecular events underlie the etiology of AF remains to be determined.

### 5.2. Alteration of the Cytoplasmic Filaments and Dysregulation of mRNA Export

Mutations in Gle1 have been described in LCCS-1 (lethal congenital contracture syndrome 1), a genetic disease characterized by a complete fetal immobility in association with neuronal and muscular defects [133]. Further analyses of a zebrafish model of Gle1 depletion revealed neurogenic and non-neurogenic developmental defects that cause LCCS-1-like phenotypes [134]. The molecular alterations caused by the Gle1 mutations linked to LCCS-1 were investigated in yeast and HeLa cells. The main homozygous mutation found in LCCS-1 cases results in the insertion of a proline-phenylalanine-glutamine (PFQ) tripeptide within the essential N-terminal coiled-coil domain of Gle1, disturbing Gle1 oligomerization at NPCs and subsequently triggering mRNA export defects [135]. Interestingly, other Gle1 mutations found in LCCS-1 or LAAHD (lethal arthrogryposis with anterior horn cell disease), a related disease, lead to delocalization of Gle1 from NPCs [136]. Defects in Gle1-dependent mRNP release at the cytoplasmic face of NPCs are therefore likely to contribute to both LCCS-1 and LAAHD diseases.

Remodeling of the cytoplasmic filaments has also been observed in another disease-related situation. The translation initiation factor eIF4E, an oncogenic protein that is upregulated in about 30% of cancers [137], is known to function as a specific mRNA export adaptor for a subset of mRNAs [7,8]. Overexpression of eIF4E alters the cytoplasmic face of the NPC by increasing Gle1 and Dbp5 levels, while reducing Nup358/RanBP2 stability and relocalizing Nup214 in the nucleoplasm [138]. This remodeling of the cytoplasmic filaments appears to somehow favor the export of eIF4E target mRNAs, although the underlying molecular mechanisms remain to be determined. Such oncogene-mediated reprogramming of the NPC and its impact on mRNA export and gene expression are expected to contribute to cellular transformation [138].

### 5.3. FG-Nucleoporins Targeted by Viruses and Inhibition of Cellular mRNA Export

DNA viruses, whose genome is transcribed in the host nucleus, and RNA viruses, which primarily replicate in the cytoplasm, have evolved multiple strategies to inhibit the export of cellular mRNAs. These mechanisms could prevent the expression of factors of innate immunity, favor the translation of viral RNAs and, in certain situations, their nuclear export [103,129]. In some cases, viruses inhibit cellular mRNA export by triggering changes in the NPC.

Notably, RNA viruses from both *Enterovirus* and *Cardiovirus* genera can block cellular mRNA export in association with the alteration of the NPC structure [139,140,141,142]. On the one hand, the protease 2A (2A^pro^) of *Enteroviruses* triggers the cleavage of Nup153, Nup98 and Nup62, precisely removing the FG-domains in the latter case ([143] and the references therein). Expression of 2A^pro^, but not of an inactive version of the protease, leads to a block of cellular mRNA export in HeLa cells [139]. On the other hand, the Leader protein (L) of *Cardioviruses* triggers hyperphosphorylation of Nup62, Nup153, Nup214 and Nup98, most likely by hijacking cellular kinases [144,145,146]. Concomitantly, expression of the L protein causes mRNA export inhibition [146]. Viruses thus impair cellular mRNA export by targeting the same subset of FG-nucleoporins, albeit with different molecular strategies. Of note, protein import is also altered in infected cells, raising the possibility that mRNA export inhibition could partially result from defective recycling of mRNP export factors [144,145,147].

At least two other RNA viruses specifically target Nup98. The M protein of members of the *Vesiculovirus* genus can inhibit bulk mRNA export [148,149]. Remarkably, the M protein can form a tripartite complex with Nup98 and Gle2 in which it occupies the RNA binding motif of Gle2 [150,151,152,153]. As the M protein is at least partially localized at the nuclear rim, formation of this complex at NPCs may compete with mRNP binding [150]. Likewise, the NS1 protein of influenza A virus can inhibit cellular mRNA export by binding to Tap-p15 and Gle2 and by altering Nup98 protein levels [71,154].

Remarkably, treatment with interferon-gamma (IFNγ), which is known to establish an anti-viral state in the cells, induces the expression of both Nup98 and Gle2 and fully reverts the mRNA export inhibition induced by *Vesiculovirus* infection or *Enterovirus* 2A^pro^ expression [139,155,156]. On the contrary, Nup98+/− or Gle2+/− mouse cells display defects in the export of mRNAs encoding immune factors and, consistently, are highly susceptible to influenza virus-mediated cell death [71]. Nup98/Gle2-dependent mRNA export is therefore critical for resistance to viral infections and appears to be a preferred target of viruses.

## 6. Conclusions

Increasing evidence suggests that mRNA export can be modulated by regulatory events targeting key players in this process at the NPC (Figure 1). As detailed above (Section 4 and Section 5), three non-exclusive types of nucleoporin modifications have been reported, or proposed, to affect mRNA export: (i) a change in protein levels (caused by variations in stability or expression); (ii) delocalization from NPCs; and (iii) modification at NPCs, including phosphorylation, cleavage or altered oligomerization. These modifications can occur in normal or pathological situations and suggest that NPCs could integrate the information arising from multiple signaling pathways. In this respect, FG-nucleoporins and the Y-complex appear as preferred targets for a number of regulatory events. In the future, it will be important not only to identify novel modifications targeting these proteins, but also to characterize the consequences on mRNA export of previously described modifications, such as Y-complex phosphorylation [109] or ubiquitination [157]. Additional studies will be required to determine the molecular consequences of such modifications on mRNP binding and translocation.

Remarkably, only a few enzymatic activities are associated with NPCs (Figure 1); yet, they all target mRNAs and/or mRNA-associated proteins. While the helicase activity of Dbp5 is crucial for mRNP remodeling and release upon export, the endonuclease activity of Swt1 appears important for mRNA QC prior to export in yeast. Of note, the SUMO-protease activity of yeast Ulp1 and the SUMO-ligase activity of mammalian Nup358/RanBP2 have both been shown to target mRNA-associated proteins [90,158]. Whether these modifications could also contribute to the regulation of mRNA export remains an open question.
